# Preliminary Comparison of Multi-scale and Multi-model Direct Inversion Algorithms for 3T MR Elastography

**DOI:** 10.2463/mrms.mp.2016-0047

**Published:** 2016-10-11

**Authors:** Kengo Yoshimitsu, Yoshinobu Shinagawa, Toshimichi Mitsufuji, Emi Mutoh, Hiroshi Urakawa, Keiko Sakamoto, Ritsuko Fujimitsu, Koichi Takano

**Affiliations:** Department of Radiology, Faculty of Medicine, Fukuoka University, Fukuoka, Japan

**Keywords:** MR elastography, inversion algorithm, multimodel direct inversion, 3T

## Abstract

**Purpose:**

To elucidate whether any differences are present in the stiffness map obtained with a multiscale direct inversion algorithm (MSDI) vs that with a multimodel direct inversion algorithm (MMDI), both qualitatively and quantitatively.

**Materials and Methods:**

The MR elastography (MRE) data of 37 consecutive patients who underwent liver MR elastography between September and October 2014 were retrospectively analyzed by using both MSDI and MMDI. Two radiologists qualitatively assessed the stiffness maps for the image quality in consensus, and the measured liver stiffness and measurable areas were quantitatively compared between MSDI and MMDI.

**Results:**

MMDI provided a stiffness map of better image quality, with comparable or slightly less artifacts. Measurable areas by MMDI (43.7 ± 17.8 cm^2^) was larger than that by MSDI (37.5 ± 14.7 cm^2^) (*P* < 0.05). Liver stiffness measured by MMDI (4.51 ± 2.32 kPa) was slightly (7%), but significantly less than that by MSDI (4.86 ± 2.44 kPa) (*P* < 0.05).

**Conclusion:**

MMDI can provide stiffness map of better image quality, and slightly lower stiffness values as compared to MSDI at 3T MRE, which radiologists should be aware of.

## Introduction

In the management of patients with chronic liver diseases and cirrhosis, the assessment of the degree of liver fibrosis is of great importance, because progressive liver fibrosis can result in portal hypertension, liver cancer, and finally patient death.^1,2^ The usefulness of shear-wave MR elastography (MRE) in assessing the pathological grades of liver fibrosis in a non-invasive fashion has been reported.^3–10^ In MRE, acoustic shear waves are transmitted to the liver, and a modified phase-contrast sequence is then applied to detect the phase shift of the protons caused by the shear waves, which are subsequently processed with an inversion algorithm.^3–10^ There has been several types of inversion algorithms reported in the literature, including local frequency estimation,^4,11,12^ multiscale direct inversion (MSDI),^2,10,13,14^ and multimodel direct inversion (MMDI).^15,16^ It is important to validate that the data obtained by these algorithms are comparable, because results obtained with one algorithm, including cutoff values or diagnostic performance, would not be applicable to those by other ones, if there is substantial difference between them. Regarding the quantitative consistency among different inversion algorithms, little clinical data has been reported so far. To our knowledge, there has been only one report comparing the results between MSDI and MMDI, in which little difference (9%) in the stiffness values of the liver measured by either method was reported using 1.5T MRE system.^16^ MMDI is a relatively new inversion algorithm, and has been reported to have several improvements over MSDI, including superior resolution, less noise, and shorter processing time.^16^

The purpose of this study is to elucidate whether liver stiffness values calculated with MSDI and MMDI are comparable at 3T MRE, and also whether any differences are present in the stiffness map (elastogram) obtained using the two algorithms.

## Materials and Methods

Out institutional review board approved this study and waivered to obtain informed consent because of its retrospective nature.

Between 2014 September 1 and October 30, 37 consecutive patients underwent gadoxetate (EOB Primovist, Bayer HealthCare, Osaka, Japan) enhanced liver MR imaging including MRE in our institute. The protocol included precontrast T_1_-weighted in- and out-of-phase images, diffusion-weighted images, dynamic-enhanced studies, T_2_-weighed images, and hepatobiliary phase images. There were 22 men and 15 women, with age ranging from 31 to 85 years old (mean 62). Among these, 8 and 17 had hepatitis B and C hepatitis/cirrhosis, and three had alcoholic liver diseases, and underwent MR imaging for the assessment of hepatocellular carcinoma; remaining nine patients had no chronic liver disease and underwent MR imaging to rule out liver focal lesions suspected on ultrasonography.

The MR equipment used was a 3T clinical unit (Discovery 750W, GE, Milwaukee, USA) along with a 32-element phased-array coil, and MRE was obtained before contrast enhancement. A 19-cm-diameter passive pneumatic driver was positioned over the center of the right rib cage at the level of the xiphoid process and attached to an acoustic waveform generator. A 60-Hz acoustic wave was propagated to the driver. A 2D spin-echo echo-planar MRE sequence (TR/TE = 1000/59, 66 × 64 matrix, 10 mm slice thickness, 80-Hz magnetization encoding gradient) acquired magnitude and phase difference wave images using 42 cm field-of-view.^17,18^ Four slices were obtained including the level of the hepatic hilum under 16-s breath-holding. Wave images and elastogram images (stiffness map) with cross-hatching marks were automatically generated on the operating console. Cross-hatching marks represent the 95% confidence threshold mask, indicating areas unsuitable for stiffness measurement.^17,18^ Inversion algorithm used for stiffness map calculation at that time was MSDI. Liver stiffness was measured by one experienced radiologist (KY) using a copy-and-paste method,^19,20^ by placing as large free-hand region-of-interests (ROI) as possible on the stiffness map, mainly in the right hepatic lobe, avoiding apparent pathologies, large vessels, areas with inadequate wave propagation (hot spot or dark spot artifacts), and cross hatching marks.^17–20^ An average of the four slices was used to represent the liver stiffness of each patient. Measured stiffness and size of ROI (mm^2^) for each slice was recorded in a separate laptop computer at the time of routine clinical practice and stiffness measurement was not repeated especially for this study.

In June 2015, a new inversion algorithm software MMDI was installed, and the digital imaging and communication in medicine (DICOM) data of MRE of the above mentioned patients were additionally analyzed, and stiffness map or elastogram with cross-hatching marks were newly generated with MMDI.

Two radiologists (KY and YS) reviewed the two stiffness maps originated from MSDI and MMDI, referring to the wave images, anatomical images, and other relevant images, including diffusion-weighted images and hepatobiliary phase images for the presence of any focal lesions, and qualitative assessment was performed in consensus, and determined which stiffness map was better than the other; namely, MSDI is worse, similar, or better, in quality, as compared to MMDI. The items assessed were overall image quality of the stiffness map, and the prominence of hot spots or dark spots artifacts within the areas without cross-hatching marks, namely measurable areas.^19,20^ Overall image quality was determined based on the homogeneity of the whole image. Hot spots and dark spots were defined as areas showing focally elevated or decreased stiffness within areas without cross-hatching marks, respectively, showing no focal abnormality in any other sequences of the protocol, possibly attributable to wave overlap or interference.^19,20^

Measurement of the liver stiffness was then repeated on the MMDI stiffness map by the same radiologist (KY) and in the same manner, as the MSDI stiffness map measurement, and quantitative assessment was performed. The items for quantitative assessment were the measurable areas, which is represented by the size of ROI in cm^2^, and the stiffness values. Measurable areas were compared between the two systems at slice basis (n = 148). Measured stiffness in kPa was compared at patient basis (n = 37).

Finally, we subclassified the liver stiffness values at patient basis obtained from both systems into 6 grades, according to the Mayo Clinic criteria^21^ (Table 1), and assessed the concordance between them.

For comparison between two parametric variables, paired *t*-test was used. For the assessment of the relationship between the two variables, Bland-Altman test and Pearson’s correlation test were applied. *P* values less than 0.05 were considered statistically significant. The software used for all statistical assessments was JMP version 11 (SAS corporation, Cary, USA).

## Results

**Fig 1.**
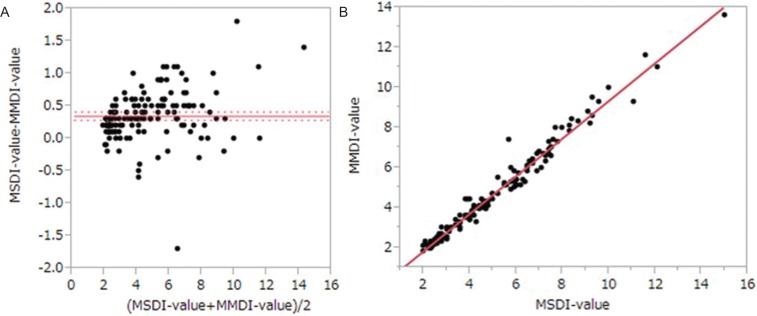
Relationship between the liver stiffness calculated with multiscale direct inversion (MSDI) and multimodel direct inversion (MMDI) algorithms. (**A**) Bland-Altman analysis. Most of the data are scattered outside of the 95% confidence interval (between 0.28 and 0.41 kPa). The dispersion of the data along y-axis is larger as the value along x-axis becomes larger. Namely, the difference between MSDI and MMDI stiffness is large, when the stiffness is large. (**B**) Regression analysis for Y, which is the MMDI stiffness, and X, which represents the MSDI stiffness. An equation, Y = −0.05 + 0.94 * X (*R*^2^ = 0.98, *P* <0.0001) was obtained, suggesting a strong and significant correlation between MSDI and MMDI stiffness.

There were 148 images in total for review (four slices per patient in 37 patients) and stiffness measurement was feasible in all slices. The measured stiffness per patient ranged from 1.9 to 15.1 kPa as measured with MSDI, and from 1.8 to 13.6 kPa as measured by MMDI, which would correspond to a full range of fibrosis grades (Fig. 1 and Table 1).

### Qualitative assessment

As for the overall image quality of stiffness maps, MSDI was worse in 103 (70%), similar in 39 (26%), and better in 6 (4%), out of 148 slices, as compared to MMDI.

As for hot spots/dark spots artifacts, MSDI had more prominent ones in 22 (15%), similar ones in 114 (77%), and less prominent artifacts in 12 (8%), out of 148 slices, as compared to MMDI.

### Quantitative assessment

Measurable areas, or the area of ROI, was 37.5 ± 14.7 cm^2^ for MSDI, and 43.7 ± 17.8 cm^2^ for MMDI, showing statistically significant difference (*P* < 0.001).

**Fig 2.**
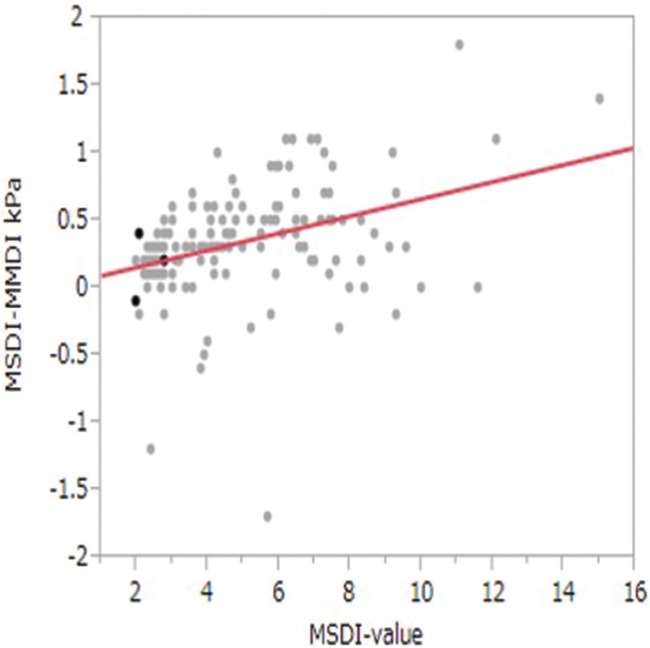
Regression analysis for Y, which is the difference in the liver stiffness calculated with multiscale direct inversion (MSDI) and multimodel direct inversion (MMDI) algorithms, and X, which represents the MSDI stiffness values. An equation, Y = 0.03 + 0.06*X (*R*^2^ = 0.1, *P* <0.0001), was obtained, suggesting a weak but significant correlation between MSDI stiffness and MSDI-MMDI difference in stiffness.

Measured stiffness values were 4.86 ± 2.44, and 4.51 ± 2.32 kPa for MSDI and MMDI, respectively, showing slight, but statistically significant differences (*P* < 0.001), and the relationship between the two are shown in Bland-Altman analysis suggests the difference in the liver stiffness between MSDI and MMDI becomes larger as the stiffness value increases (Fig. 1A and B), which was confirmed when the relationship between the difference between them and MSDI stiffness values are assessed by regression analysis (Fig. 2).

### Stiffness grades subclassification

The numbers of the patients subclassified into each fibrosis grade based on the two algorithms are shown in Table 1, along with the criteria. The results are comparable, however, three patients were graded differently between the two systems: in two of them, stiffness was changed from grade 2 to grade 1 (from 2.7 to 2.2 kPa, and from 2.6 to 2.3 kPa), and in one patient, from grade 4 to 3 (from 4.1 to 3.6 kPa), with MSDI and MMDI, respectively.

**Fig 3.**
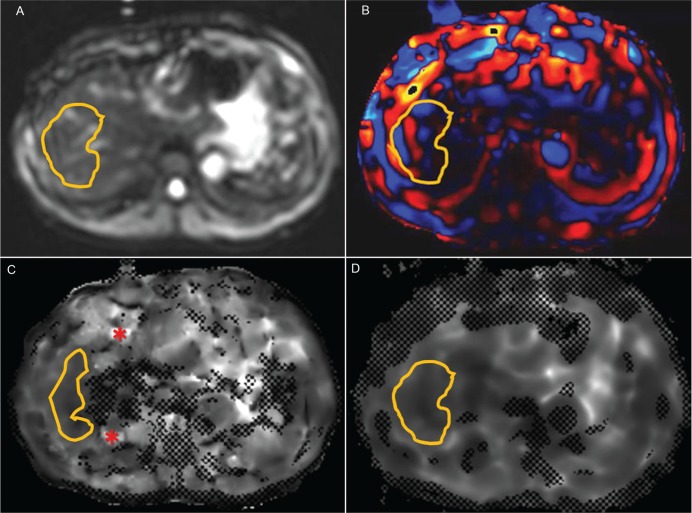
39 year-old woman with no known liver disease. (**A**) Magnitude echo-planar image. A free-hand region-of-interest (ROI) was drawn within the contour of the right lobe, avoiding major vessels and focal lesions (**B**) Wave image. Relatively good wave propagation is noted within the area of ROI (**C**) Stiffness map calculated from multiscale direct inversion algorithm. Note more cross-hatching marks, prominent “hot spots” (*), and inhomogeneity in the areas within the contour of the liver, as compared to 3D. Because of the presence of cross-hatching marks, the size of ROI needed to be made smaller than those on other images (3A–D). Stiffness value was measured to be 2.3 kPa, and subclassified as grade 1 stiffness (**D**) Stiffness map calculated from multimodel direct inversion algorithm. Note less cross-hatching marks and homogeneity in the areas within the contour of the liver, as compared to 3C. Overall image quality was assessed as better than 3C. An ROI of the same size as 3A and 3B was able to be placed. Stiffness value was measured to be 2.1 kPa, and subclassified as grade 1 stiffness.

A representative case is shown in Fig. 3.

## Disccusion

In this study, the same DICOM data of 37 patients were analyzed by two different inversion algorithms, namely MSDI and MMDI, and our results suggested MMDI would provide liver stiffness map of better image quality, comparable or slightly less artifacts, and more measurable areas, as compared to MSDI. In addition, the stiffness values of the liver obtained by MMDI are comparable, but slightly lower than that by MSDI (MMDI stiffness = −0.05 + 0.94 * MSDI stiffness, Fig. 1). The mean difference in the stiffness values was approximately 7%, ranging from −1.7 to 1.8 kPa, which are consistent with the recently reported 1.5T MRE data.^16^ Our data also suggested that the difference is proportional to the value of the stiffness, namely, the difference would be small when the measured value is small, and become larger when the value is large (Figs. 1 and 2). Because the range of the cutoff values or criteria of assessing liver fibrosis is smaller for the early stage fibrosis and larger for higher stage fibrosis (Table 1), smaller difference in stiffness between MSDI and MMDI at the early stage fibrosis may less likely cause discrepancy in the suspected liver fibrosis grade between MSDI and MMDI. Actually, we confirmed that only three patients turned out to be down-graded in term of suspected fibrosis grade by applying MMDI, as compared to MSDI. However, care should be taken particularly when assessing patients longitudinally, for example, who are under anti-viral therapy, because the measured stiffness of the liver obtained with these two different inversion algorithms may lead to erroneous interpretation of the therapeutic effects.

One of the limitation in this study is small number of patients (n = 37) assessed, however, the number of slices (images) were relatively large (n = 148), and the measured stiffness ranged widely enough to cover the whole fibrosis grades (Table 1). We therefore consider our data are reliable. A second limitation is that the items we assessed, namely overall image quality, degree of artifacts, and size of ROI, may all affect each other, at least to some extent, and therefore these are not independent parameters. Other limitation may include the way we performed qualitative assessment. Two radiologists assessed the images in consensus, but ideally, assessment should have been done independently. Lack of pathological correlation might be listed as another limitation, but we consider that is out of the scope of this study.

In conclusion, MMDI can provide elastograms or stiffness maps of better image quality as compared to MSDI at 3T MRE, and the stiffness values obtained by MMDI is slightly (7%) lower than those obtained by MSDI. Radiologists should be aware of this issue, particularly when patients are assessed longitudinally using these two different inversion algorithms.

## Figures and Tables

**Table 1.** Stiffness grades according to Mayo Clinic criteria^21^ and number of patients classified into each grade by each algorithm

	Stiffness	Fibrosis grades	MSDI	MMDI
Grade 1	<2.5 kPa	normal	7	9
Grade 2	2.5–2.9 kPa	normal or inflammation	5	3
Grade 3	2.9–3.5 kPa	stage 1 to 2	1	2
Grade 4	3.5–4.0 kPa	stage 2 to 3	5	4
Grade 5	4.0–5.0 kPa	stage 3 to 4	5	5
Grade 6	>5.0 kPa	stage 4 or cirrhosis	14	14

Stage 1–4: fibrosis grade according to Metavir system.^22,23^ MSDI: multiscale direct inversion, MMDI: multimodel direct inversion.
